# Differential Expression of Vegfr-2 and Its Soluble Form in Preeclampsia

**DOI:** 10.1371/journal.pone.0033475

**Published:** 2012-03-12

**Authors:** Carine Munaut, Sophie Lorquet, Christel Pequeux, Capucine Coulon, Jeanne Le Goarant, Frédéric Chantraine, Agnès Noël, Frédéric Goffin, Vassilis Tsatsaris, Damien Subtil, Jean-Michel Foidart

**Affiliations:** 1 Laboratory of Tumor and Development Biology, CHU, GIGA-Cancer, University of Liège, Liège, Belgium; 2 Department of Obstetrics and Gynecology, Hôpital la Citadelle, Liège, Belgium; 3 Maternité Jeanne de Flandre, CHRU, Lille, France; 4 Maternité Port-Royal, Hôpital Cochin–Saint-Vincent-de-Paul, AP-HP, Paris, France; Brigham and Women's Hospital, United States of America

## Abstract

**Background:**

Several studies have suggested that the main features of preeclampsia (PE) are consequences of endothelial dysfunction related to excess circulating anti-angiogenic factors, most notably, soluble sVEGFR-1 (also known as sFlt-1) and soluble endoglin (sEng), as well as to decreased PlGF. Recently, soluble VEGF type 2 receptor (sVEGFR-2) has emerged as a crucial regulator of lymphangiogenesis. To date, however, there is a paucity of information on the changes of VEGFR-2 that occur during the clinical onset of PE. Therefore, the aim of our study was to characterize the plasma levels of VEGFR-2 in PE patients and to perform VEGFR-2 immunolocalization in placenta.

**Methodology/Principal findings:**

By ELISA, we observed that the VEGFR-2 plasma levels were reduced during PE compared with normal gestational age matched pregnancies, whereas the VEGFR-1 and Eng plasma levels were increased. The dramatic drop in the VEGFR-1 levels shortly after delivery confirmed its placental origin. In contrast, the plasma levels of Eng and VEGFR-2 decreased only moderately during the early postpartum period. An RT-PCR analysis showed that the relative levels of VEGFR-1, sVEGFR-1 and Eng mRNA were increased in the placentas of women with severe PE. The relative levels of VEGFR-2 mRNA as well as expressing cells, were similar in both groups. We also made the novel finding that a recently described alternatively spliced VEGFR-2 mRNA variant was present at lower relative levels in the preeclamptic placentas.

**Conclusions/Significance:**

Our results indicate that the plasma levels of anti-angiogenic factors, particularly VEGFR-1 and VEGFR-2, behave in different ways after delivery. The rapid decrease in plasma VEGFR-1 levels appears to be a consequence of the delivery of the placenta. The persistent circulating levels of VEGFR-2 suggest a maternal endothelial origin of this peptide. The decreased VEGFR-2 plasma levels in preeclamptic women may serve as a marker of endothelial dysfunction.

## Introduction

Preeclampsia (PE), a syndrome affecting 2–5% of pregnancies, is a leading cause of maternal and fetal morbidity and mortality in developed countries [1]. This pregnancy-specific syndrome is characterized by the onset of hypertension and proteinuria later than 20 weeks of gestation [2]. Although PE is a multifactorial disease, recent data indicate that imbalances between pro-angiogenic factors, such as vascular endothelial growth factor (VEGF) and placental growth factor (PIGF), and anti-angiogenic factors that inhibit angiogenesis, such as soluble vascular endothelial growth factor receptor 1 (sVEGFR-1) and soluble endoglin (sEng), are closely related to the pathogenesis of the disease [3], [4], [5], [6].

The VEGFRs, VEGFR-1 and VEGFR-2, are structurally related members of the tyrosine kinase receptor family that mediate critical signaling pathways in endothelial cells [7]. The kinase activity of VEGFR-1 is low compared to that of VEGFR-2 [8]. Whereas VEGFR-1 is considered to be a “decoy” receptor, VEGFR-2 is regarded as the major mediator of the broad signaling cascades that regulate many endothelial cell functions, including proliferation, migration and differentiation [9], [10]. VEGF and its receptors (VEGFR-1 and VEGFR-2, as well as their soluble forms) are expressed in the placental trophoblasts throughout gestation [11].

Soluble VEGFR-1, also known as soluble fms-like tyrosine kinase 1 (sFlt-1), displays anti-VEGF and anti-PlGF activities and is therefore a robust anti-angiogenic factor. It is increased prior to the clinical onset of the disease in women with PE. The soluble VEGFR-1 levels correlate with the severity and the time to the onset of the disease [12], [13]. Endoglin (Eng or CD105), a cell surface co-receptor for the β1 and β3 isoforms of transforming growth factor (TGF), is highly expressed in endothelial cells and trophoblasts [14]. TGF-β3, which is strongly expressed during early placentation (6–8 weeks) and decreases at the end of the first trimester (10–12 weeks), inhibits trophoblast migration and invasion. In pregnancies complicated by early-onset preeclampsia, TGF-β3 expression remains abnormally elevated and trophoblast differentiation is arrested at an intermediate immature phenotype stage [15]. The TGF-β1 levels are lower and the levels of soluble endoglin (sEng), a truncated form of Eng, are higher in pregnant women who subsequently develop preeclampsia than in healthy pregnant women [16]. Circulating Eng and VEGFR-1 may synergize and contribute to the PE syndrome through distinct but additive mechanisms.

Whereas the role of the splice variant sVEGFR-1 in PE is well established (see [17], [18] for reviews), less is known about sVEGFR-2. Soluble VEGFR-2 was first reported in mouse and human plasma [19] and initially described as resulting from an endothelial cell surface proteolytic cleavage following the ligand-induced down expression of VEGFR-2 [20], [21]. Recently, alternative splicing of the VEGFR-2 gene has been reported in mice [22]. The identified splice variant is an endogenous VEGF-C antagonist and a crucial regulator of lymphatic vessel growth. The implications of this splice variant for PE have not been determined. Circulating soluble VEGFR-2 in the plasma of patients has been reported [19], [20]. Although VEGFR-2 is expressed in the placenta [23], there is a paucity of information about the implications for PE pathogenesis of its circulating soluble form and of the recently described spliced variant. This study was therefore undertaken to evaluate plasma VEGFR-2 concentrations, the relative placental mRNA expression and the plasma levels after delivery in normal pregnancies and those complicated by preeclampsia in comparison to sVEGFR-1 and endoglin.

## Results

### Demographics

The clinical characteristics of the patients in the preeclamptic (PE) and control (NP) groups are presented in Table 1. The gestational ages at delivery and the median birth weights of the neonates were significantly lower in the preeclamptic group than in the control group. The majority of the preeclamptic patients exhibited severe proteinuria (>3 g/24 h), elevated blood pressure (BP) (≥160 mm Hg systolic BP and ≥100 mm Hg diastolic BP) and low platelet counts (<100,000/mm^3^). Abnormal umbilical and uterine Doppler results were found in 19 of the 30 preeclamptic patients.

**Table 1:** Clinical characteristics.

	NP		PE	
	Inclusion	Delivery	Inclusion	Delivery
N	15	15	12	30
Maternal age (mean)	29.5±4.3	28.2±4.6	29±5	30±5
Parity	2±1	2±1	1±1*	1±1*
Primiparity	4 (27%)	6 (39%)	7 (63%)	21 (70%)
Gestational age (weeks)	30±4	38±2	29±4	32±3**
BMI (kg/m^2^)	21.6±4.4	22.7±5.4	23.5±3.7	24.4±4.2
Neonatal Weight (g)	NA	3057±468	NA	1466±508**
Proteinuria>3 gr/24 h	0	0	7 (59%)	18 (60%)**

NP: normal pregnancies; PE: severe preeclampsia; NA: not applicable; BMI: body mass index.

* *P*<0.05 and.

** *P*<0.001 compared to NP group.

### Evolution of the circulating VEGFR-2, VEGFR-1 and Eng in the maternal plasma

**Figure 1:**
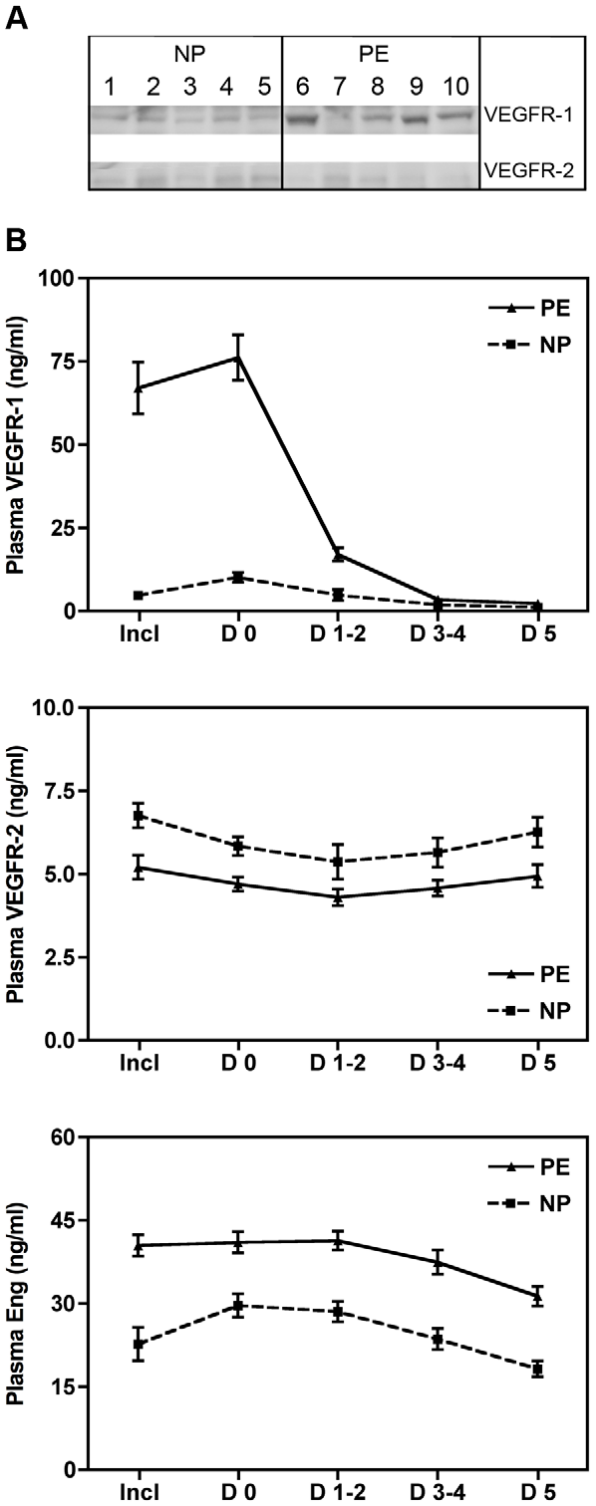
Maternal plasma analysis. **A**: Western blot analysis of VEGFR-1 and VEGFR-2 from gestational age-matched normal pregnant women (NP, 1–5) and preeclamptic women (PE, 6–10). **B**: Maternal plasma concentration of VEGFR1, VEGFR2 and Eng (mean±SE, pg/ml) at inclusion (Incl), delivery (day 0, D 0) and during post partum (day 1 to day 5, D 1 to D 5). PE: severe preeclampsia (plain line), NP: normal pregnancies (dashed line).

The maternal plasma concentrations of VEGFR-1, VEGFR-2 and Eng were evaluated in both groups (Table 2). At delivery, the plasma concentration of VEGFR-2 in the preeclamptic group was significantly lower than that in the healthy, gestational-age-matched controls (4.41 (3.93–5.27) vs. 6,54 (5.51–8.18) ng/ml, *P*<0.0001). In contrast, the plasma levels of both VEGFR-1 and Eng increased in the preeclamptic patients compared with the gestational-age-matched controls (66.01 (46.62–100.87) vs. 4.79 (2.21–6.08) ng/ml, *P*<0.0001 and 43.13 (31.01–51.70) vs. 21.59 (15.22–27.74) ng/ml, *P* = 0.0003). Therefore, both groups displayed inverse associations between the circulating concentrations of VEGFR-2 and those of VEGFR-1 and Eng. Western blot further confirmed the presence of the same immunoreactive bands for VEGFR-1 and VEGFR-2 in the maternal circulation of the preeclamptic women and the gestational-age matched controls (Figure 1A). During the early postpartum period (Figure 1B), the plasma VEGFR-2 levels were stable in the preeclamptic (*P* = 0.2184) and control pregnancy (*P* = 0.2743) groups, although a rapid and significant VEGFR-1 decrease was detected in the plasma of the preeclamptic patients (*P*<0.0001) and the controls (*P*<0.0001). A moderate (20%) decrease in the circulating Eng was measured 5 days after delivery in the preeclamptic patients (*P* = 0.0074) and the controls (*P* = 0.0045).

**Table 2:** Maternal plasma concentration of VEGFR-1, VEGFR-2 and Eng in normal gestational age matched to PE delivery and preeclamptic pregnancies (median and quartiles (25^th^–75^th^ centile), ng/ml).

	NP	PE	*P* value
VEGFR-1	4.79 (2.21–6.08)	66.01 (46.62–100.87)	*P*<0.0001
VEGFR-2	6.54 (5.51–8.18)	4.41 (3.93–5.27)	*P*<0.0001
Eng	21.59 (15.22–27.74)	43.13 (31.01–51.70)	*P* = 0.0003

### Relative mRNA quantification in the placentas

**Figure 2:**
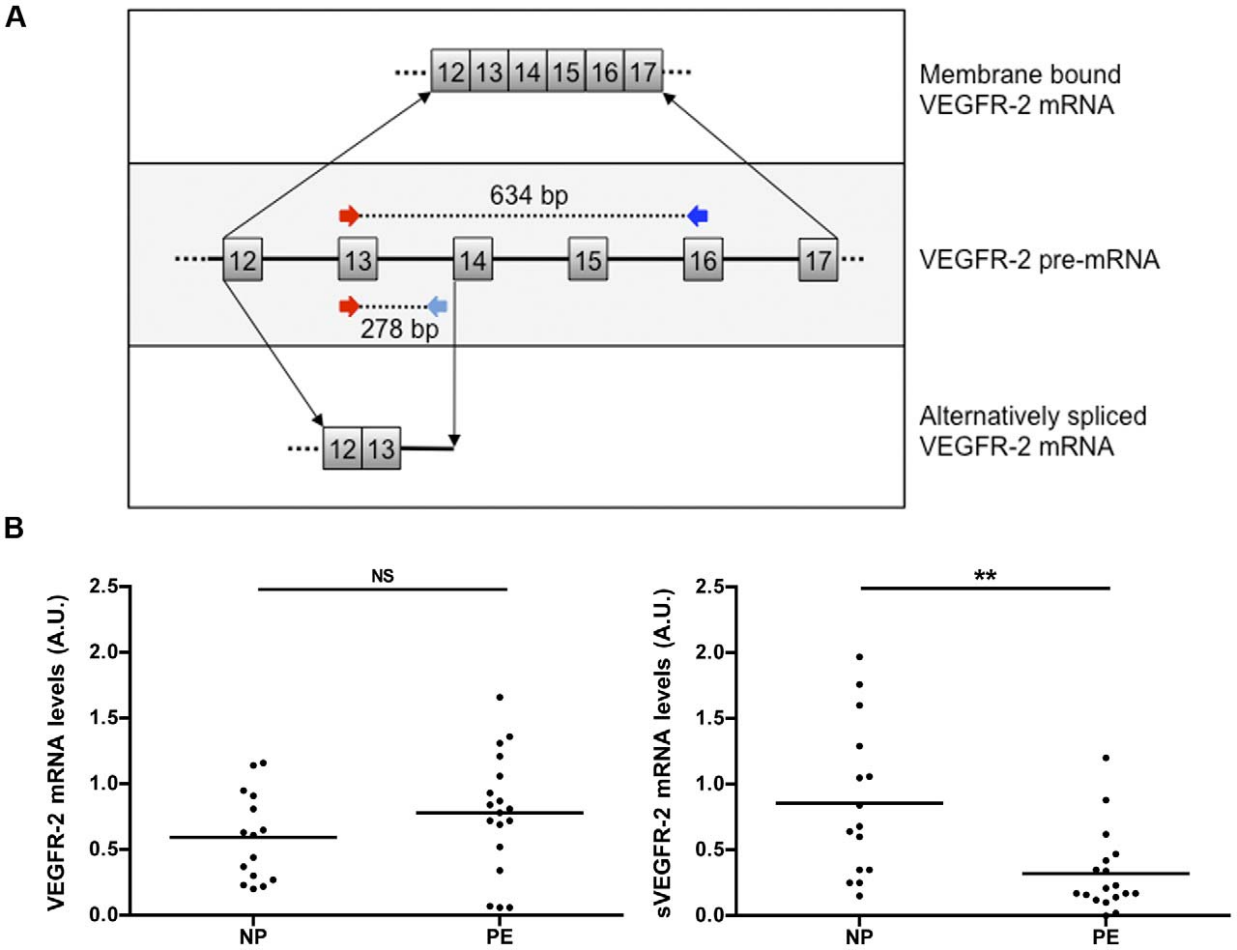
Placental VEGFR-2 and sVEGFR-2 relative mRNA levels. **A**: Schematic representation of pre-mRNA exon-intron structure of VEGFR-2 (central shadowed box not at scale) with primers used to discriminate membrane bound VEGFR-2 mRNA (upper box, exon 13-exon 16; 634 bp) and soluble VEGFR-2 mRNA (lower box, exon 13-intron 13; 278 bp). **B**: Relative mRNA levels are expressed as arbitrary units (A.U.). NP: normal pregnancies, PE: severe preeclampsia. **P<0.001 compared to NP group; ns: non-significant compared to NP group.

The murine corneal splice variant of the VEGFR-2 gene, which encodes a secreted form of the protein [22], retains intron 13 and contains an in-frame termination codon. The resulting protein lacks the transmembrane and intracellular tyrosine kinase domains of the membrane-bound VEGFR-2. To search for a similar soluble splicing variant in the human placenta, we devised primers targeting human exon 13, intron 13 and exon 16 (Table 3 and Figure 2 A). A PCR using the exon 13 and intron 13 primers yielded a 278-bp product that spanned the location of the splicing event, whereas a PCR using the exon 13 and exon 16 primers yielded a 634-bp product that corresponded to the membrane-bound VEGFR-2.

**Table 3:** Sequence of primers and TaqMan probes used for RT-PCR studies.

Gene (Accession N°)	Sequence
**VEGFR1-FP**	5′-TCCCTTATGATGCCAGCAAGT-3′
**VEGFR1-RP**	5′-CCAAAAGCCCCTCTTCCAA-3′
**VEGFR1 Probe**	5′-CCGGGAGAGACTTAAACTGGGCAAATCA-3′
AF063657	
**sVEGFR1-FP**	5′-ACAATCAGAGGTGAGCACTGCAA-3′
**sVEGFR1-RP**	5′-TCCGAGCCTGAAAGTTAGCAA-3′
**sVEGFR1 Probe**	5′-TCCAAATTTAAAAGCACAAGGAATGATTGTACCAC-3′
U01134	
**VEGFR2-FP**	5′-CTTGGCCCACAGCCTCTGCC-3′
**VEGFR2-RP**	5′-GTTCCCCTCCATTGGCCCGC-3′
NM_002253	
**sVEGFR2-FP**	5′-CTTGGCCCACAGCCTCTGCC-3′
**sVEGFR2-RP**	5′-GGCATTCCAACTGCCTCTGCAC-3′
NM_002253**+**NT_022853	
**ENG-FP**	5′-TTTGTCTTGCGCAGTGCTTACT-3′
**ENG-RP**	5′-TTTTCCGCTGTGGTGATGA -3′
**ENG Probe**	5′-TGAGGCGGTGGTCAATATCCTGTCGA-3′
NM_000118	

Using those specific primers, we found that the sVEGFR-2 mRNA splice variant was less abundant in the placentas of the preeclamptic women than in those of the controls, although the membrane-bound specific VEGFR-2 mRNA levels were similar between the two groups (Figure 2 B).

**Figure 3:**
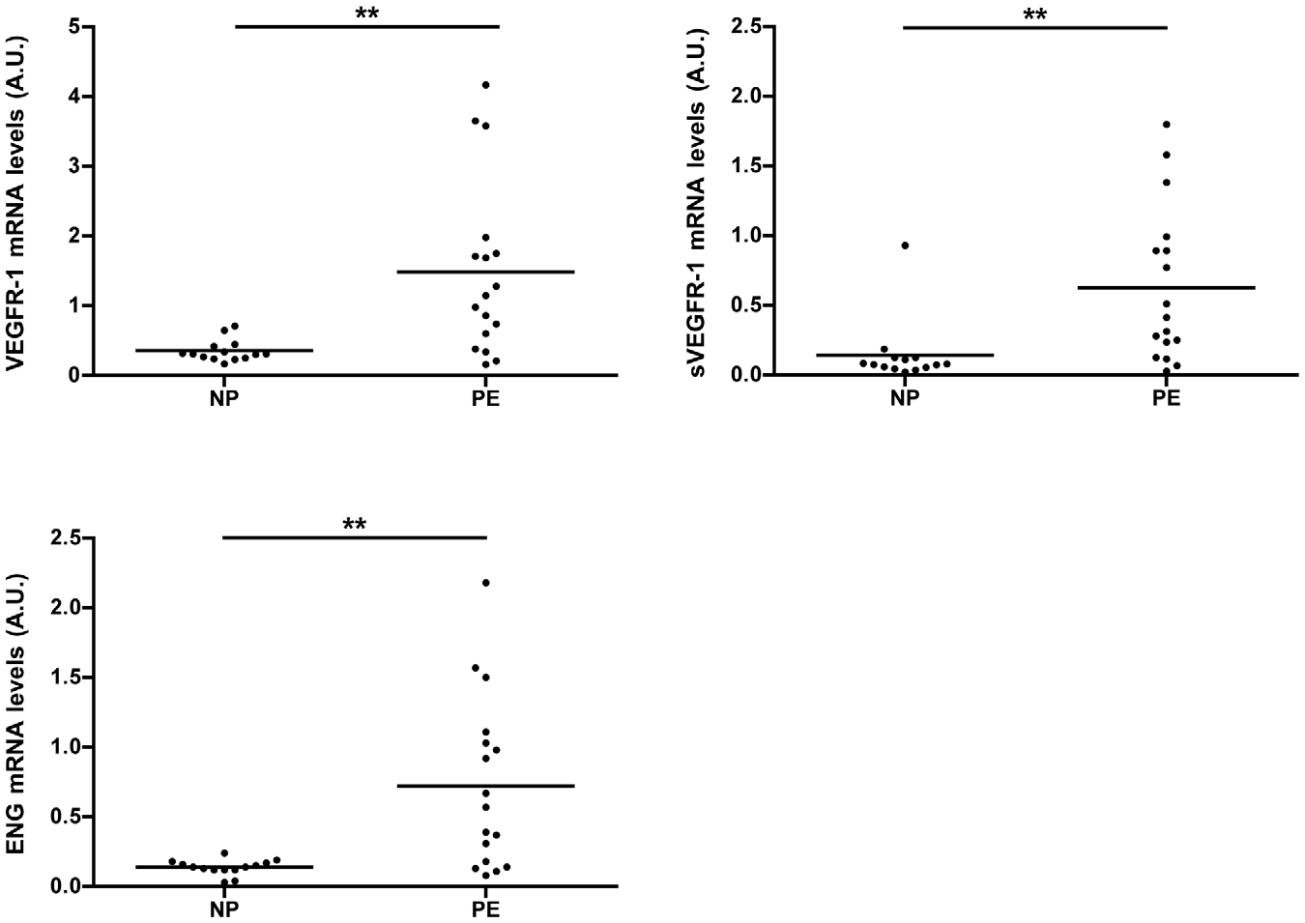
Placental VEGFR-1, sVEGFR-1 and Eng relative mRNA levels. Relative mRNA levels are expressed as arbitrary units (A.U.). NP: normal pregnancies, PE: severe preeclampsia. **P<0.001 compared to NP group; ns: non-significant compared to NP group.

The relative levels mRNA of VEGFR-1, sVEGFR-1 and endoglin were higher in the pregnancies complicated by preeclampsia (Figure 3).

### Immunolocalization of VEGFR-1 and VEGFR-2

**Figure 4:**
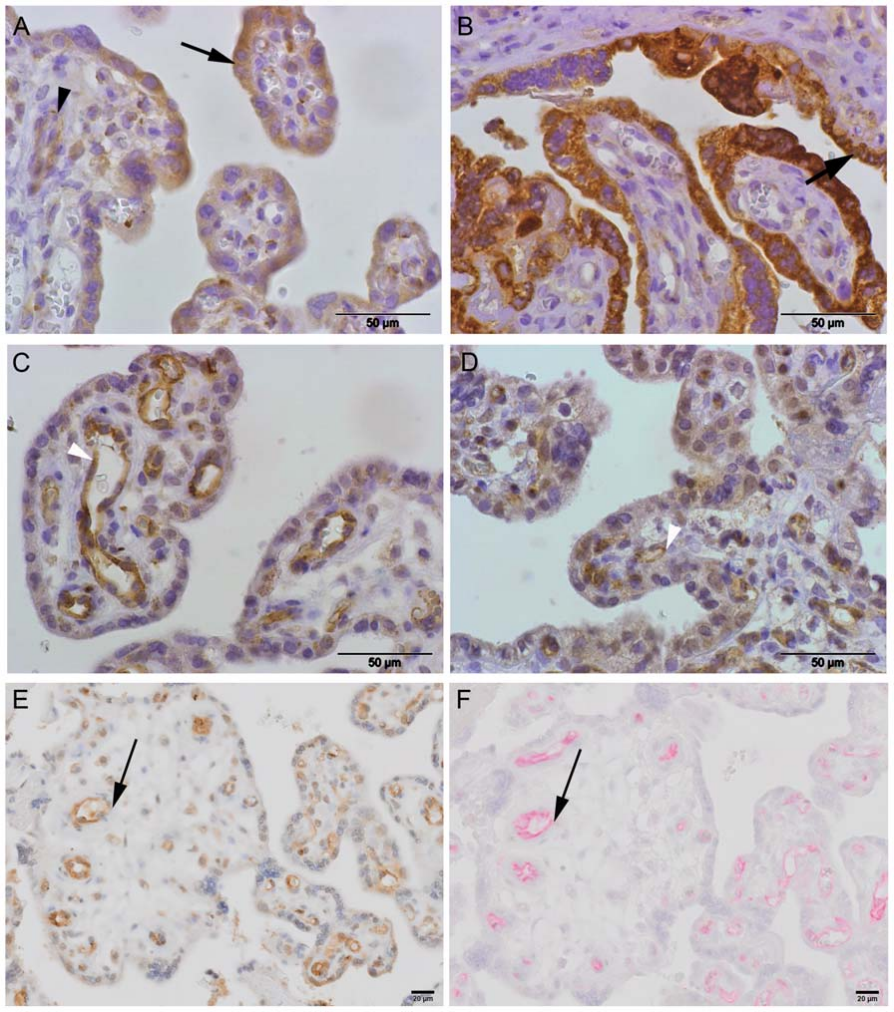
Representative immunolocalization of VEGFR-1 (A–B), VEGFR-2 (C, D and E) and CD31 (F). Placental villi from a normal pregnant women (A, C, E and F) and from a preeclamptic women (B–D): VEGFR-1 is expressed in the cytotrophoblasts, syncytiotrophoblasts (arrow) and also in some endothelial cells (white arrow head). VEGFR-2 is mainly localized in vascular endothelial cells (arrows) in placentas of both groups as identified by serial section immunostaining with CD31.

The cells that produce VEGFR-1 and VEGFR-2 in the placenta were identified with immunohistochemistry. VEGFR-1 was primarily detected in the cytotrophoblasts, syncytiotrophoblasts, endothelial cells and some stromal cells of the placentas from normal pregnancies. A similar but more intense staining pattern was observed in the same cell types of the placentas from the preeclamptic women (Figure 4 A and B). VEGFR-2 was mainly localized in the vascular endothelial cells of both the normal and preeclamptic placental villi as revealed by serial section staining with the CD31, blood vessel specific marker (Figure 4 C–F and Figure S1).

## Discussion

Consistent with previous reports [3], [5], [6], [24], the circulating plasma concentrations of VEGFR-1 and Eng were higher in the PE than in the control group in this study (Figure 1, *P*<0.0001 and *P* = 0.0004, respectively). The soluble VEGFR-2 was identified as a 160-kDa immunoreactive band by western blot (Figure 1A), as also reported by Ebos *et al.* [19] and Albuquerque *et al.* [22]. The results of the ELISA analysis indicated that this soluble VEGFR-2 isoform was present at reduced levels in the circulation of the preeclamptic women. Other recent research has documented that VEGFR-2 plasma levels decreased during early preeclampsia or in pregnancies with intrauterine growth retardation [25], [26], [27]. Chaiworapongsa *et al.* also documented that the mean plasma concentrations of VEGFR-2 are identical in nonpregnant women (n = 40) and in those experiencing normal pregnancies (n = 135) [26]. Similarly, we did not find any differences in the VEGFR-2 concentrations between 10 nonpregnant women and 15 women with normal pregnancies (data not shown). Collectively, these data indicate that preeclampsia is associated with decreased plasma concentrations of VEGFR-2, whereas normal pregnancy is not. Our study is the first to find that significantly reduced plasma concentrations of VEGFR-2 in PE patients are associated with decreased placental expression of the sVEGFR-2 mRNA splice variant (Figure 2 B).

The persistently lower plasma VEGFR-2 levels observed in postpartum PE women (Figure 1) suggest that both decreased placental expression of the VEGFR2 spliced variant and maternal endothelial dysfunction affect the plasma VEGFR2 levels. Although the membrane-bound isoform of VEGFR-2 is primarily expressed in activated maternal endothelial cells, a subset of the circulating maternal hematopoietic cells, the CD34+ endothelial progenitor cells, also express VEGFR-2 [28]. Therefore, maternal endothelial dysfunction and CD34+ alterations associated with PE may contribute to the decreased plasma levels of VEGFR-2 associated with this condition.

After placental delivery, the plasma VEGFR-1 levels in the preeclamptic women rapidly returned to the levels observed in normotensive women, as would be expected for a trophoblast-derived peptide. In contrast, the circulating plasma levels of VEGFR-2 in the PE patients remained stable and lower than in the normotensive women during the first 5 days after delivery, and the Eng plasma levels remained stable and higher. The stability of the circulating plasma levels of Eng and VEGFR-2 suggests that these two peptides arise from both maternal sources and trophoblasts.

Whereas VEGFR-2 is expressed on cytotrophoblast stem cells and on cells in the proximal columns of the chorionic villi during the first and second trimesters of pregnancy [29], at delivery VEGFR-2 was localized almost exclusively to the placental endothelial cells, in contrast to VEGFR-1 that is expressed mainly by trophoblasts [11], [23].

The affinity of VEGF for VEGFR-2 is at least 10-fold lower than its affinity for VEGFR-1. Because the plasma VEGFR-2 levels are decreased by a maximum of 20%, this decrease is probably insufficient to compensate for the VEGF inhibition elicited by the approximately 10-fold increase in plasma VEGFR-1.

We have previously reported that the relative expression of VEGFR-2 mRNA in preeclamptic placental villi was similar to that in normal placentas [5]. In that study, the total VEGFR-2 mRNA was measured using RT-PCR without discriminating between the membrane-bound and soluble forms. Using specific primers to quantify the mRNA levels of the full-length VEGFR-2 and its splice variant in the present study, we found that the relative mRNA levels of the sVEGFR-2 splice variant are lower in the placentas of the preeclamptic women than in those of the controls, although the relative levels of membrane-bound VEGFR-2 mRNA are similar.

During the postpartum period, the circulating plasma levels of VEGFR-2 remained lower in the PE patients than in the women with normal pregnancies. Because VEGFR-2 is the major mediator of the effects of VEGF, we hypothesize that the persistently lower plasma concentrations of VEGFR-2 in preeclamptic women result from a decrease in the availability of free VEGF to stimulate VEGFR-2 synthesis and trafficking at the endothelial cell surfaces, as shown in other models [30]. The low circulating plasma levels of VEGFR-2 observed in the preeclamptic women may also reflect a low regenerative capacity in endothelial cells. Indeed, preeclampsia is associated with an endothelial dysfunction [31], [32] that is characterized by increased endothelial cell-mediated vasoconstriction, increased vascular permeability and increased endothelium-mediated platelet aggregation. These effects may lead to maternal hypertension and proteinuria. Some researchers have found that in women with a history of preeclampsia, impaired endothelial function may be an indicator of cardiovascular risk [32]. Such women exhibit impaired endothelial function for up to 1 year postpartum [33]. The concept of maternal endothelial recovery is further supported by the 5-fold increase in circulating endothelial cells in preeclampsia patients compared with the healthy controls. The number of these cells declines rapidly after delivery, in parallel with the clinical recovery of the patient [34]. The functional manifestations of glomerular endothelial cell injury also largely resolve within the first postpartum month [35]. The normalization of endothelial vasoactive substances that precedes clinical recovery in preeclamptic women after delivery further strengthens the concept of postpartum endothelial recovery [36]. Altogether, our data indicate that the plasma levels of anti-angiogenic factors particularly VEGFR-2, VEGFR-1 and sEng behave in different ways in the early postpartum period in preeclamptic women. The persistently low level of circulating VEGFR-2 is associated with the continued high concentration of soluble Eng and the rapid drop in circulating placenta-derived VEGFR-1. The decreased VEGFR-2 plasma levels in preeclamptic women may serve as a marker of endothelial dysfunction.

## Materials and Methods

### Sample collection

Samples were collected from pregnant women recruited from the department of Obstetrics and Gynecology at La Citadelle Hospital, Liège, Belgium, and from the Maternité Jeanne de Flandre, CHRU, Lille, France. The local ethics committees approved this study, and written informed consent was obtained from all the patients.

Thirty subjects had severe preeclampsia (PE), which was defined using the hypertension, edema and proteinuria criteria. Hypertension was defined as systolic and diastolic blood pressures above 160 mmHg and 100 mmHg, respectively, in at least two consecutive measurements spaced 4 hours apart. Severe proteinuria was defined as more than 300 mg per 24 hours or a urine sample with proteinuria of at least 3+. Thirty patients with non-complicated pregnancies (NP) constituted the control group for the plasma measurements. None of the patients had chronic hypertension, renal diseases or endocrine diseases. Among the control group, blood was collected from 15 gestational-age-matched to PE delivery. All the blood samples (PE and NP) were collected in tubes containing EDTA. The samples were centrifuged at 3000 rpm during 20 minutes at 4°C. The plasma aliquots were stored at −80°C until analysis.

The placenta samples were collected during the deliveries by cesarean section (NP = 15 and PE = 18). The tissue samples were frozen in liquid nitrogen within 30 minutes after the birth and stored at −80°C until further analysis. Maternal plasma was additionally collected between postpartum days 1 and 5.

### Immunoassays

The plasma levels of VEGFR-1, VEGFR-2 and Eng were measured with an enzyme-linked immunoassay (ELISA) technique using DuoSet® human immunoassays (R&D Systems Europe Ltd., Abington, UK). The lower limits of detection of the assays were 15 pg/ml for VEGFR-1 and 5 pg/ml for VEGFR-2 and ENG. All the samples were measured in duplicate. The samples with duplicate coefficients of variation exceeding 15% were reanalyzed.

For western blotting the maternal plasma (diluted 1∶20, 20 µl/lane) was separated by 8.5% SDS-PAGE and transferred onto polyvinylidene difluoride (PVDF, Immobilon™-P, Millipore, Billerica, MA, USA) membranes. After activation in 100% methanol, the blots were incubated for 1 h at room temperature in TBS-T/casein (TBS-T (Tris-HCl 20 mM, NaCl 140 mM, Tween® 20 0.2%, pH 7.6) containing 1% casein) (Sigma-Aldrich, St Louis, MO, USA). After being washed, the membranes were incubated overnight at 4°C with either the VEGFR-2 antibody (AF357, diluted 1∶500, R&D Systems) or the VEGFR-1 antibody (AF321, diluted 1∶500, R&D Systems) in TBS-T containing 1% BSA. After being washed, the membranes were incubated for 1 h at room temperature with the appropriate secondary HRP-linked antibody diluted in TBS-T/BSA (P0449, Dako Cytomation) and were washed again. The immunocomplexes were visualized via a chemiluminescence reaction (Pierce® ECL Western Blotting Substrate, Thermo Scientific, Rockford, IL, USA) on a luminescent image analyzer (LAS-4000, Fujifilm, Wavre, Belgium).

### RT-PCR analysis

The placenta tissue biopsies from the 18 PE and 15 NP women were immediately snap frozen and stored in liquid nitrogen until the analysis. The frozen tissues were processed as previously described [5]. For real-time quantitative RT-PCR analyses gene-specific primers and TaqMan fluorescent hybridization probes (Eurogentec, Liège, Belgium) for the VEGFR-1, sVEGFR-1 and ENG mRNAs were used (Table 3). Primers sequences were deposit in a public database (RTPrimerDB: http://www.rtprimerdb.org/). The PCR was performed using the ABI PRISM 7700 Sequence Detection System instrument and software (PE Applied Biosystems, Inc., Foster City, CA). The expression level of each gene of interest was computed relative to that of 18S rRNA (Predeveloped TaqMan Assay, PE Applied Biosystems) to normalize the variations in the RNA quality and in the amount of input cDNA, as previously described [5]. All the primers, except those specific for VEGFR2, were designed to produce fragments that spanned the exon-intron boundaries, thus preventing the amplification of genomic DNA. The sVEGFR-2 reverse primer recognized an intron 13 motif that is specific to the truncated transcript variant of the secreted form of VEGFR-2 [22]. The RT-PCR products specific for the VEGFR-2 and sVEGFR-2 mRNAs were measured in 10-ng aliquots of cDNA using Taq polymerase (Takara, Shiga, Japan) and 5 pmol of each primer (Table 3). The PCR products were resolved on 10% polyacrylamide gels (Bio-Rad) and analyzed with a luminescent image analyzer (LAS-4000, Fujifilm) after GelStar staining (Lonza Rockland, Inc., Rockland, ME). The specific mRNA levels were expressed as the ratios of the specific transcripts to the 18S rRNA.

### Immunohistochemistry

The placentas were fixed in 4% formalin for 4 to 12 hours, embedded in paraffin and cut into 4-µm sections. The sections were mounted on SuperFrost Plus glass slides (Menzel-Gläser, LaboNord), dewaxed in xylene, rehydrated and subsequently autoclaved for 11 min at 126°C and 1.4 bar in Target Retrieval Buffer (S2031, DakoCytomation, Glostrup, Denmark). The endogenous peroxidases were blocked by treatment with 3% H_2_O_2_/H_2_O for 20 min, and non-specific binding was prevented by incubation in Universal Blocking Reagent (BioGenex, San Ramon, CA, USA) for 3 min. The sections were incubated overnight at 4°C with the primary antibody (VEGFR-1, R&D Systems, AF321, diluted 1∶300; VEGFR-2, R&D Systems, AF357, diluted 1∶100, CD-31, Dako, M0823, diluted 1∶40). The sections were washed in TBS (Tris-buffered saline) with 0.1% Tween and then incubated for 30 min with a secondary antibody (biotinylated rabbit anti-goat (Dako E0466, diluted 1∶400) or biotinylated goat anti-mouse (Dako K0433, diluted 1∶400)), followed by incubation with peroxidase-labeled streptavidin for 30 min (Dako P0397, diluted 1∶500). The staining was detected with DAB chromogen after 5 min or with permanent red substrate (Dako K0640) for CD31. The nuclei were counterstained by incubation with hematoxylin for 2 min. The sections were mounted, examined and photographed. The control samples were processed by omitting the primary antibody or by incubating the sections with nonspecific IgG at the same concentration as the primary antibody.

### Statistical analyses

The patient groups were compared using the Kruskal-Wallis test, and significant differences were further analyzed via pairwise comparisons using the Mann-Whitney test. The results are presented as medians±quartiles (25^th^–75^th^ quartiles) or means±SE. *P* values<0.05 were considered statistically significant.

## Supporting Information

Figure S1 **Representative immunolocalization of, αSMA (A), VEGFR-2 (B) and CD31 (C) in serial sections of normal placental villi.** Double immunofluorescences of αSMA (green) and VEGFR-2 (red) (D–F) or CD31 (green) and VEGFR-2 (red) (G–I). Nuclei are counterstained with DAPI (blue).
(DOCX)
