# Supplementary figures and images for: Differential Expression of Vegfr-2 and Its Soluble Form in Preeclampsia
Source: PLoS One. 2012 Mar 12;7(3):e33475. doi: 10.1371/journal.pone.0033475 (PMC3299790; doi:10.1371/journal.pone.0033475)

Figure S1


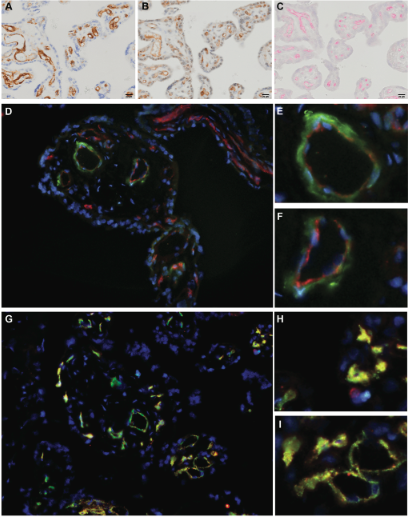

Supplement: Figure S1 — Representative immunolocalization of, αSMA (A), VEGFR-2 (B) and CD31 (C) in serial sections of normal placental villi. Double immunofluorescences of αSMA (green) and VEGFR-2 (red) (D–F) or CD31 (green) and VEGFR-2 (red) (G–I). Nuclei are counterstained with DAPI (blue). (DOCX) [file pone.0033475.s001.docx]
